# Why Are Children in Urban Neighborhoods at Increased Risk for Psychotic Symptoms? Findings From a UK Longitudinal Cohort Study

**DOI:** 10.1093/schbul/sbw052

**Published:** 2016-05-06

**Authors:** Joanne Newbury, Louise Arseneault, Avshalom Caspi, Terrie E. Moffitt, Candice L. Odgers, Helen L. Fisher

**Affiliations:** ^1^MRC Social, Genetic & Developmental Psychiatry Centre, Institute of Psychiatry, Psychology & Neuroscience, King’s College London, London, UK;; ^2^Departments of Psychology and Neuroscience, Psychiatry and Behavioral Sciences, and Centre for Genomic and Computational Biology, Duke University, Durham, NC;; ^3^Center for Child and Family Policy and the Sanford School of Public Policy, Duke University, Durham, NC

**Keywords:** childhood psychotic symptoms, neighborhood characteristics, social cohesion, psychosis, urbanicity

## Abstract

**Background:**

Urban upbringing is associated with a 2-fold adulthood psychosis risk, and this association replicates for childhood psychotic symptoms. No study has investigated whether specific features of urban neighborhoods increase children’s risk for psychotic symptoms, despite these early psychotic phenomena elevating risk for schizophrenia and other psychiatric disorders in adulthood.

**Methods:**

Analyses were conducted on over 2000 children from the Environmental Risk (E-Risk) Longitudinal Twin Study, a nationally-representative cohort of UK-born twins. Neighborhood-level characteristics were assessed for each family via: a geodemographic discriminator indexing neighborhood-level deprivation, postal surveys of over 5000 residents living alongside the children, and in-home interviews with the children’s mothers. Children were interviewed about psychotic symptoms at age 12. Analyses were adjusted for important family-level confounders including socioeconomic status (SES), psychiatric history, and maternal psychosis.

**Results:**

Urban residency at age-5 (OR = 1.80, 95% CI = 1.16–2.77) and age-12 (OR = 1.76, 95% CI = 1.15–2.69) were both significantly associated with childhood psychotic symptoms, but not with age-12 anxiety, depression, or antisocial behavior. The association was not attributable to family SES, family psychiatric history, or maternal psychosis, each implicated in childhood mental health. Low social cohesion, together with crime victimization in the neighborhood explained nearly a quarter of the association between urbanicity and childhood psychotic symptoms after considering family-level confounders.

**Conclusions:**

Low social cohesion and crime victimization in the neighborhood partly explain why children in cities have an elevated risk of developing psychotic symptoms. Greater understanding of the mechanisms leading from neighborhood-level exposures to psychotic symptoms could help target interventions for emerging childhood psychotic symptoms.

## Introduction

Urban vs rural upbringing doubles a child’s odds of developing schizophrenia in adulthood.^1^ The association between urbanicity and psychosis has been frequently replicated,^2–10^ shows a degree of specificity to non-affective psychoses,^4,7,11,12^ and is not explained by a range of potential confounding factors^2,13–15^ including migration of individuals with schizophrenia into cities.^16^ These converging lines of evidence suggest that the association between urbanicity and psychosis has genuine aetiological underpinnings,^16–19^ though the mechanisms driving the association are currently unknown. Urbanicity is therefore a key area for psychosis research, considering that over two-thirds of the world’s population are predicted to live in cities by 2050.^20,21^

The vast majority of urbanicity-psychosis research has focused on adult psychosis. Yet urban residency from birth to adolescence, rather than during adulthood, appears to be more strongly associated with adult psychosis.^6,10,13^ Consistent with the neurodevelopmental model of schizophrenia, this suggests that the processes leading from urban exposure to psychosis begin in adolescence, childhood, or earlier. Notably, positive psychotic symptoms, such as hallucinations and delusions, are surprisingly prevalent among children in the general population.^22–26^ These early psychotic phenomena share familial and environmental risk factors with psychotic disorders,^27–29^ and whilst they are usually transitory,^22,23,30^ children who experience psychotic symptoms have a significantly elevated risk for schizophrenia and other psychoses in adulthood.^31,32^ Additionally, childhood psychotic symptoms have broad psychiatric relevance as they significantly heighten risk for other subsequent mental health difficulties including substance abuse,^26^ depression,^26^ PTSD,^32^ and suicidal behavior.^32,33^ Childhood psychotic symptoms are therefore a useful marker of early-life risk indicators for psychosis and general psychopathology. Childhood psychotic symptoms could also shed light on the urbanicity-psychosis association: a handful of studies have shown that these symptoms occur more frequently^29^ and are more likely to persist into adulthood among youth living in urban vs nonurban settings.^34,35^ However, no studies have tested whether specific aspects of the urban environment increase risk for psychotic symptoms among children.

Indeed, urbanicity is only a proxy for the currently unknown operative risk factor(s) for psychosis.^18,36^ More recently, attention has turned to potential urban characteristics^37^ operating at the neighborhood-level. Neighborhood-level deprivation has been frequently implicated in adult psychosis.^17,38–43^ However, modern urban neighborhoods are very mixed in terms of poverty and affluence,^44^ whilst adult psychosis risk increases incrementally through increasing levels of urbanicity.^2,3,6,13^ Furthermore, the association between urbanicity and psychosis appears stronger in more recent generations,^9,45^ despite urban populations becoming generally wealthier. Thus, the association is difficult to explain through neighborhood-level deprivation alone. Cumulative evidence also supports the importance of neighborhood-level social processes such as crime,^40,46^ disorganization^46,47^ and social fragmentation^37,39,41,48^ in adult psychosis (thoroughly reviewed by March et al^36^), which are purported to increase adult psychosis risk by heightening childhood exposure to social stressors.^18,19,49,50^ Intriguingly, prodromal status among young adults has been shown to follow spatial patterning in accordance with these kinds of neighborhood-level psychosocial characteristics.^51^ However, the longitudinal associations between neighborhood-level social processes and childhood psychotic symptoms are currently unknown. Ultimately, such research could help target social and clinical interventions for early psychotic symptoms.

**Fig. 1.**
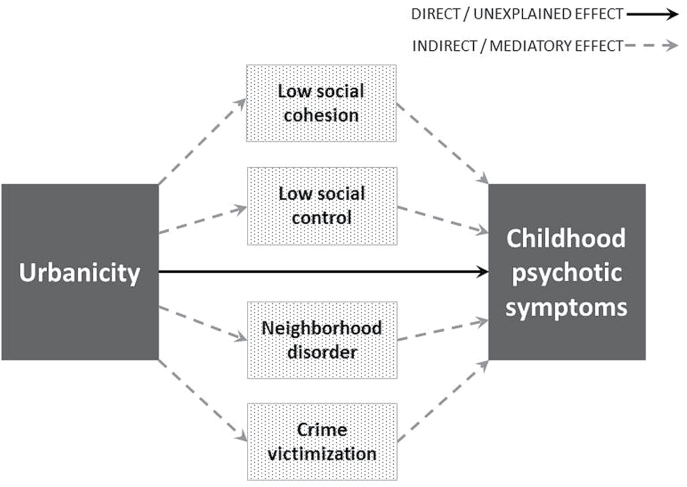
Conceptualized pathways between urbanicity and childhood psychotic symptoms, with the total effects transmitting both directly (solid line), and indirectly (dashed lines) via neighborhood-level social process mediators (low social cohesion, low social control, high neighborhood disorder, and high crime victimization).

Here we draw from sociological theory and evidence illustrating that neighborhood-level social processes mediate the effect of neighborhood structural features (eg, urbanicity) on a range of health outcomes.^52–54^ Guided by this theory and adult psychosis findings, the current study focuses on 4 neighborhood-level social processes: (1) social cohesion, describing the cohesiveness and supportiveness of relationships between neighbors^52^; (2) social control, describing the likelihood that neighbors would intervene in problems in the neighborhood^52^; (3) neighborhood disorder, describing physical and social evidence of disorder/threat within the neighborhood^53^; and (4) crime victimization, representing more direct experiences of victimization in the neighborhood (eg, mugging). The current study investigates the pathways leading from urbanicity to childhood psychotic symptoms, whilst differentiating the effects of specific neighborhood-level social processes from family-level effects. We utilized a cohort of 2232 nationally-representative British twin children who have been followed from birth to age 12 and interviewed for psychotic symptoms at age 12. Our longitudinal neighborhood-level measures were obtained from multiple sources, and neighborhood scores were allocated with fine geographic resolution (ie, postcode-level). With these measures, we asked: (1) Are children in urban vs nonurban neighborhoods at increased risk for psychotic symptoms? (2) Is this association specific to childhood psychotic symptoms? (3) Is the association between urbanicity and childhood psychotic symptoms explained by background characteristics of families living in cities? (4) Are urban neighborhoods more likely to lack social cohesion and social control and be characterized by disorder and crime? (5) Finally, does the level of social cohesion, social control, neighborhood disorder, and crime victimization operating within neighborhoods mediate the effect of urban residency on childhood psychotic symptoms? We hypothesized that the effect of urbanicity on childhood psychotic symptoms would be specific to this phenotype, and mediated via exposure to low social cohesion and social control, and high disorder and crime victimization in the neighborhood (proposed pathways shown in figure 1).

## Methods

### Study Cohort

Participants were members of the Environmental Risk (E-Risk) Longitudinal Twin Study, which tracks the development of a nationally-representative birth cohort of 2232 British twin children. The sample was drawn from a larger cohort of twins born in England and Wales in 1994–1995.^55^ Full details about the sample are reported elsewhere.^56^ Briefly, the E-Risk sample was constructed in 1999–2000, when 1116 families with same-sex 5-year-old twins (93% of those eligible) participated in home-visit assessments. Families were recruited to represent the UK population of families with newborns in the 1990s, based on residential location throughout England and Wales and mothers’ age (teenaged mothers with twins were over-selected to replace high-risk families who were selectively lost to the register through non-response. Older mothers having twins via assisted reproduction were under-selected to avoid an excess of well-educated older mothers). E-Risk families are representative of UK households across the spectrum of neighborhood-level deprivation: 25.6% of E-Risk families live in “wealthy achiever” neighborhoods compared to 25.3% of households nation-wide; 5.3% vs 11.6% live in “urban prosperity” neighborhoods; 29.6% vs 26.9% live in “comfortably off” neighborhoods; 13.4% vs 13.9% live in “moderate means” neighborhoods; and 26.1% vs 20.7% live in “hard-pressed” neighborhoods.^57,58^ E-Risk families underrepresent “urban prosperity” neighborhoods because such households are likely to be childless. Sex was evenly distributed in the resulting sample (49% male). All families were English speaking, and the majority (93.7%) were White. Follow-up home-visits were conducted when children were aged 7, 10, and 12 (participation rates were 98%, 96%, and 96%, respectively). At age 12, the E-Risk sample comprised 2146 twin children, and the majority of these children had complete data on both psychotic symptoms and urbanicity at age 12 (95.7%; *N* = 2054). Over half of children (56.7%, *N* = 1180) never moved house at all between ages 5 and 12, and of those who did nearly two-thirds (65.0%) moved less than 500 meters. The Joint South London and Maudsley and the Institute of Psychiatry Research Ethics Committee approved each phase of the study. Parents gave informed consent and children gave assent.

### Measures

#### Childhood Psychotic Symptoms.

E-Risk families were visited by mental health trainees or professionals when children were aged 12.^29^ Each child was privately interviewed about 7 psychotic symptoms pertaining to delusions and hallucinations, with items including “have other people ever read your thoughts?,” “have you ever thought you were being followed or spied on?,” and “have you ever heard voices that other people cannot hear?.” This interview has been described in detail previously.^29^ The item choice was guided by the Dunedin Study’s age-11 interview protocol^31^ and an instrument prepared for the Avon Longitudinal Study of Parents and Children.^59^ Interviewers coded each experience 0, 1, 2 indicating respectively “not a symptom,” “probable symptom,” and “definite symptom.” A conservative approach was taken in designating a child’s report as a symptom. First, the interviewer probed using standard prompts designed to discriminate between experiences that were plausible (eg, “I was followed by a man after school”) and potential symptoms (eg, “I was followed by an angel who guards my spirit”), and wrote down the child’s narrative description of the experience. Second, items and interviewer notes were assessed by a psychiatrist expert in schizophrenia, a psychologist expert in interviewing children, and a child and adolescent psychiatrist to verify the validity of the symptoms. Third, because children were twins, experiences limited to the twin relationship (eg, “My twin and I often know what each other are thinking”) were coded as “not a symptom”. Children were only designated as experiencing psychotic symptoms if they reported at least one definite symptom. At age 12, 5.9% (*N* = 125) of children reported experiencing psychotic symptoms. This is similar to the prevalence of psychotic symptoms in other community samples of children and adolescents.^22–26^ Furthermore, we previously showed that childhood psychotic symptoms in this cohort have good construct validity, sharing many of the genetic, social, neurodevelopmental, and behavioral risk factors and correlates as adult schizophrenia.^29^ Additionally, as we focused on psychotic symptoms rather than diagnoses, the present study design avoids confounding by psychiatric service utilization.

#### Urbanicity.

Urban/nonurban classification of E-Risk families’ neighborhoods was based on responses from a postal survey sent to residents living alongside E-Risk families when children were aged 12.^60,61^ Questionnaires were sent to every household in the same postcode as the E-Risk families, excluding the E-Risk families themselves (addresses were identified from electoral roll records). The number of surveys sent ranged from 15 to 50 residences per neighborhood (Average = 18.96, SE = 0.21). Excluding undelivered surveys (*N* = 600), the overall response rate was 28.1% (5601/19 926). Survey respondents typically lived on the same street or within the same apartment block as the children in our study. Surveys were returned by an average of 5.18 (SD = 2.73) respondents per neighborhood (range = 0–18 respondents), and there were at least 2 responses from 95% of the neighborhoods (*N* = 5601 respondents).^61^ Residents reported whether their neighborhood was in “a city,” “a town,” “a suburb,” “a small village,” or “the countryside.”^29^ There was high agreement between residents in the same neighborhood, with only 50 neighborhoods returning discordant responses (ie, neighborhoods where residents differed in their urbanicity responses). These 50 ambiguous neighborhoods were clarified by a British researcher (blind to any phenotypic/identifying data) using the children’s full postcode, Google Aerial view and the Office of National Statistics’ population density map (http://www.neighbourhood.statistics.gov.uk/HTMLDocs/PopulationDensity_2010.html, last accessed April 28, 2016), based on a combination of features including population density, building density, proximity to the countryside or city/town centre, land-use (eg, agriculture, transportation, industry, etc.), and the official definition of the settlement. This same method was used to estimate urbanicity at age-5 for the 35% of children who had moved over 500 metres between ages 5 and 12. For ease of interpretation and to increase analytic power, urbanicity is herein dichotomized as urban (1: city/town) vs nonurban (0: suburb/small village/countryside). At age 12, the sample was split evenly between urban and nonurban neighborhoods, with 51.9% (*N* = 1066) of children living in urban neighborhoods and the remaining 988 children living in nonurban neighborhoods. Similarly, 55.1% (*N* = 1117) lived in urban neighborhoods at age 5.

#### Neighborhood-Level Deprivation.

Neighborhood-level deprivation was constructed using A Classification of Residential Neighbourhoods (ACORN), a geodemographic discriminator developed by CACI Information Services (http://www.caci.co.uk/, last accessed April 28, 2016).^57^ Detailed information about ACORN’s classification of neighborhood-level socioeconomic-status (SES) has been provided previously.^58,60,62^ Briefly, CACI utilized over 400 variables from 2001 census data for Great Britain (eg, educational qualifications, unemployment, housing tenure) and CACI’s consumer lifestyle database. Following hierarchical-cluster-analysis, 5 distinct and homogeneous ordinal groups were created ranging from “Wealthy Achiever” (coded 1) to “Hard Pressed” (coded 5) neighborhoods. Each family in our sample was matched to the ACORN code for its neighborhood via its postcode (age 5 or age 12 postcode, where relevant).^58^

#### Neighborhood-Level Social Processes.

Social processes included social cohesion, social control, neighborhood disorder and crime victimization, and were measured in both early and late childhood. Social processes were first measured at age 5 via in-home interviews with the children’s mothers.^63^ Social cohesion^52^ (5 items) was assessed by asking mothers whether their neighborhood was close-knit, whether neighbors shared values, and whether neighbors trusted and got along with each other, etc. Higher scores indicate greater social cohesion. Social control^52^ (5 items) was assessed by asking mothers to judge whether people in their neighborhoods would take action against different types of undesirable activities (eg, children skipping school, fights in public places). Higher scores indicate greater social control. For neighborhood disorder,^53^ mothers were asked whether 13 problems affected their neighborhood, including noisy neighbors, arguments or loud parties, vandalism, graffiti or deliberate damage to property, and cars broken into. Higher scores indicate greater neighborhood disorder. Crime victimization was assessed by asking mothers whether they or their family had been victimized by violent crime (eg, mugging, assault), a burglary, or a theft in the neighborhood. Higher scores indicate greater crime victimization. Items (each coded 0–2) within each social process scale were summed for each mother. Social processes were also measured when children were aged 12 via the resident surveys^60,61^ (survey methodology described in detail under urbanicity heading). Residents were asked the same questions regarding these 4 neighborhood-level social processes. For the resident reports, the social process scales were created in 2 steps. First, items belonging to each social process scale were averaged to create summary scores for each of the 5601 respondents. Second, scores for each E-Risk family were created by averaging the social process scores of respondents within that neighborhood.

Thus, neighborhood-level social processes were estimated both before and contemporaneously to childhood psychotic symptoms, enabling us to triangulate a prospective design with objective neighborhood appraisals. At age 5, mothers’ views of the neighborhood were used as mothers are considered more reliable reporters than children at this age and because their perceptions are likely to influence their children’s amount of exposure and experiences in the neighborhood.^64,65^ At age 12, resident reports were used to gain more objective and comprehensive assessments of the neighborhood. As children themselves reported on their own psychotic symptoms at age 12, both our age-5 (mother-reported) and age-12 (resident-reported) assessments of neighborhood-level social processes are obtained from independent sources.

#### Other Age-12 Outcomes.

Anxiety was assessed when children were aged 12, via private interviews using the 10-item version of the Multidimensional Anxiety Scale for Children (MASC).^66^ An extreme anxiety group was formed with children who scored at or above the 95th percentile (*N* = 129, 6.1%). Depression symptoms were assessed at age 12 using the Children’s Depression Inventory (CDI).^67^ Children who scored 20 or more^68^ were deemed to have clinically significant depressive symptoms (*N* = 74, 3.5%). Antisocial behavior was assessed using the Achenbach system of empirically-based assessment.^69^ An extreme antisocial behavior group was formed with children who scored at or above the 95th percentile (*N* = 110, 5.1%), based on combined mother and teacher reports at age 12.^70^

#### Family-Level Confounders.

Family SES was measured via a composite of parental income (total household), education (highest mother/father), and occupation (highest mother/father) when children were aged 5, and was categorized into tertiles (ie, low-, medium-, and high-SES). Family psychiatric history and maternal psychosis were both assessed when children were aged 12. In private interviews, mothers reported on family history of DSM disorders,^71^ which was converted to a proportion (0–1.0) of family members with a history of psychiatric disorder. For maternal psychosis, mothers were interviewed using the Diagnostic Interview Schedule^72^ for DSM-IV^73^ which provides a symptom count for characteristic symptoms of schizophrenia (eg, hallucinations, delusions, anhedonia).

#### Statistical Analysis.

Analyses were conducted in STATA 11.2 (Stata-Corp). Firstly, linear regression was used to investigate the association between urbanicity and neighborhood-level social processes (table 1). Secondly, logistic regression was used to investigate the associations between neighborhood-level social processes and childhood psychotic symptoms (table 2). Thirdly, our mediation analyses utilized KHB pathway decomposition (table 3).^74^ This procedure partitions the total effect of one variable (urbanicity) on another variable (childhood psychotic symptoms) into the direct effect (which also includes the effects of unknown/unspecified mediators and measurement error), and indirect effects explained by specified mediators (neighborhood-level social processes). Age-5 urbanicity is used when age-5 social processes are analyzed; age-12 urbanicity is used when age-12 social processes are analyzed. As the scales differed between the age-5 (mother-reported) and age-12 (resident-reported) social process variables, social process variables in steps 2 and 3 were standardized with a mean of 0 and a SD of 1 (subtraction of the mean then division by the SD) to facilitate comparability of the results. Where appropriate, analyses accounted for the nonindependence of observations using the “CLUSTER” command because the sample comprised twins. This procedure is derived from the Huber-White variance estimator, and provides robust standard errors adjusted for within-cluster correlated data^75^ (Note: within-pair twin correlations can also be corrected using multi-level approaches. Supplementary table 1 shows that our main logistic regression analyses are highly robust to alternative estimation procedures.).

**Table 1.** Bivariate Associations Between Urbanicity and Neighborhood-Level Social Processes

Neighborhood-Level Social Processes	Range	Urban	Nonurban	Standardized Association Between Urbanicity and Social Processes	
		*M* (SD)	*M* (SD)	*B* ^a^	*P* Value
Age-5 (mother reports)^b^					
Social cohesion	0–10	7.11 (2.95)	8.18 (2.32)	−.19	<.001
Social control	0–10	7.04 (2.88)	7.91 (2.41)	−.16	<.001
Neighborhood disorder	0–22	4.40 (4.15)	3.46 (3.24)	.12	<.001
Crime victimization	0–6	1.06 (1.39)	0.75 (1.18)	.12	<.001
Age-12 (resident reports)^c^					
Social cohesion	0–4	2.11 (0.50)	2.36 (0.47)	−.25	<.001
Social control	0–4	2.09 (0.53)	2.33 (0.51)	−.22	<.001
Neighborhood disorder	0–2	0.56 (0.35)	0.40 (0.32)	.23	<.001
Crime victimization	0–2	0.22 (0.24)	0.15 (0.19)	.16	<.001

*Note*: *B*, standardized beta coefficient; *M*, mean. Social cohesion and social control consistently have negative beta coefficients, demonstrating that urban neighborhoods had lower levels of social cohesion and social control compared to nonurban neighborhoods. In contrast, neighborhood disorder and crime victimization consistently have positive beta coefficients, demonstrating that urban neighborhoods had higher levels of disorder and crime victimization compared to nonurban neighborhoods. All analyses account for the nonindependence of twin observations.

^a^The standardized (*B*) beta coefficients indicate the unit SD change in each social process given 1 unit SD change in urbanicity, and allow comparison between each social process. Standardized betas provide exactly the same point estimates as correlation coefficients and may be interpreted as correlations, with a score of −1.0 indicating a 100% negative correlation and a score of +1.0 indicating a 100% positive correlation.

^b^Age-5 urbanicity is used for the bivariate associations between urbanicity and age-5 mother-reported social processes.

^c^Age-12 urbanicity is used for the bivariate associations between urbanicity and age-12 resident-reported social processes. Age-12 resident-reported social process scores were imputed for 2 children with missing data.

**Table 2.** Bivariate Associations Between Neighborhood-Level Social Processes and Childhood Psychotic Symptoms

Neighborhood-Level Social Processes	OR	95% CI	*P* Value
Age-5 (mother reports)			
Social cohesion	0.68	[0.58, 0.82]	<.001
Social control	0.75	[0.62, 0.91]	.003
Neighborhood disorder	1.26	[1.06, 1.51]	.010
Crime victimization	1.40	[1.19, 1.65]	<.001
Age-12 (resident reports)^a^			
Social cohesion	0.76	[0.65, 0.89]	.001
Social control	0.83	[0.69, 1.00]	.050
Neighborhood disorder	1.27	[1.07, 1.52]	.007
Crime victimization	1.17	[0.96, 1.42]	.123

*Note*: Social cohesion and social control are consistently associated with odds lower than 1 for childhood psychotic symptoms, demonstrating that children were less likely to experience psychotic symptoms in neighborhoods with higher levels of social cohesion and social control. In contrast, neighborhood disorder and crime victimization are consistently associated with odds greater than 1 for childhood psychotic symptoms, demonstrating that children were more likely to experience psychotic symptoms in neighborhoods with higher levels of neighborhood disorder and crime victimization. All analyses account for the nonindependence of twin observations. All social process variables have been standardized with a mean of 0 and a SD of 1.

^a^Age-12 resident-reported social process scores were imputed for 2 children with missing data.

**Table 3.** Association Between Urbanicity and Childhood Psychotic Symptoms, Split Into Total Effects, and Direct and Indirect Pathways via Neighborhood-Level Social Process Mediators

Potential Neighborhood-Level Social Process Mediators	Mediation Model 1					Mediation Model 2^a^				
	Sample Size	Total OR [95% CI]	Direct OR [95% CI]	Indirect OR [95% CI]	% Mediated^b^	Sample Size	Total OR [95% CI]	Direct OR [95% CI]	Indirect OR [95% CI]	% Mediated^b^
Age-5 (mother reports)^c^										
Social cohesion	2014	1.71* [1.10, 2.64]	1.49^†^ [0.95, 2.35]	1.15** [1.05, 1.25]	**25**	2005	1.58* [1.00, 2.49]	1.46 [0.92, 2.33]	1.08* [1.02, 1.16]	**17**
Social control	1998	1.74* [1.13, 2.70]	1.62* [1.04, 2.54]	1.07* [1.00, 1.15]	**13**	1989	1.62* [1.03, 2.55]	1.57^†^ [0.99, 2.48]	1.04 [0.99, 1.09]	8
Neighborhood disorder	2022	1.72* [1.11, 2.66]	1.65* [1.06, 2.57]	1.04 [0.99, 1.09]	7	2013	1.58* [1.01, 2.49]	1.57^†^ [0.99, 2.47]	1.01 [0.98, 1.04]	2
Crime victimization	2022	1.71* [1.11, 2.65]	1.59* [1.02, 2.48]	1.07* [1.02, 1.13]	**13**	2013	1.57^†^ [1.00, 2.47]	1.50^†^ [0.95, 2.37]	**1.05 [1.00, 1.10]**	**11**
Age-12 (resident reports)^d^										
Social cohesion	2054	1.76** [1.15, 2.69]	1.58* [1.03, 2.43]	1.11* [1.02, 1.21]	**19**	2045	1.61* [1.03, 2.50]	1.53^†^ [0.98, 2.40]	1.05 [0.97, 1.13]	10
Social control	2054	1.76** [1.15, 2.69]	1.66* [1.07, 2.57]	1.06 [0.97, 1.16]	11	2045	1.61* [1.03, 2.51]	1.58* [1.00, 2.50]	1.02 [0.94, 1.10]	4
Neighborhood disorder	2054	1.76** [1.15, 2.69]	1.60* [1.01, 2.53]	1.10^†^ [1.00, 1.21]	16	2045	1.61* [1.03, 2.51]	1.55^†^ [0.97, 2.49]	1.04 [0.96, 1.12]	7

*Note*: The sample sizes and total effect ORs vary slightly for the age-5 mother reports of social processes, due to small numbers of children missing data on neighborhood-level social processes and/or family-level covariates. These sample size differences also account for the difference between the main effect OR (1.80) and the total effect ORs for mother reports in Mediation model 1. Social processes can still be compared for the percentage that they mediate the total effect of urbanicity. We also conducted mediation analyses using Full Information Maximum Likelihood (FIML) in Mplus to include all available cases at age 5 (*N* = 2232 for all Models reported) and found no differences in the size, direction or pattern of effects. Total effect = overall association between urbanicity and childhood psychotic symptoms; direct effect = the part of the overall association that is not explained by the mediator/covariates in the model; and indirect effect = the part of the overall association that is explained by the social process mediator in the model. Bold text denotes significant indirect (mediation) pathways at *P* < .05. All analyses account for the nonindependence of twin observations. All social process variables have been standardized with a mean of 0 and a SD of 1.

^a^The total, direct and indirect ORs in mediation model 2 are adjusted for family-level confounders: family socioeconomic status, family psychiatric history, and maternal psychosis.

^b^Percentages rounded to whole numbers.

^c^Age-5 urbanicity is used for the mediation analysis of urbanicity and childhood psychotic symptoms via age-5 mother-reported social processes.

^d^Age-12 urbanicity is used for the mediation analysis of urbanicity and childhood psychotic symptoms via age-12 resident-reported social processes. Age-12 resident-reported social process scores were imputed for 2 children with missing data.

*P < .05, **P < .01, ^†^Nominally significant *P* > .05 and *P* < .1.

## Results

### Are Children in Urban vs Nonurban Neighborhoods at Increased Risk for Psychotic Symptoms?

There was a significant cross-sectional association between age-12 urban residency and childhood psychotic symptoms (OR = 1.76, 95% CI = 1.15–2.69, *P* = .009). Around 7.4% (*N* = 79) of urban-dwelling children compared to 4.4% (*N* = 43) of nonurban-dwelling children experienced at least one definite psychotic symptom at age 12. The association between urbanicity and psychotic symptoms held when analyses were restricted to the 56.6% of children who never moved house between ages 5 and 12 (OR = 2.01, 95% CI = 1.14–3.58, *P* = .017), and when controlling for residential mobility during this period (OR = 1.71, 95% CI = 1.12–2.61, *P* = .014). The association also held for the 93.7% of children who were ethnically White (OR = 1.85, 95% CI = 1.21–2.84, *P* = .005). Although in our sample there was a tendency for urban neighborhoods to be more deprived (OR = 2.57, 95% CI = 1.99–3.32, *P* < .001), half of urban neighborhoods were relatively affluent (ACORN categories 1–3; 50.3%), and over a quarter of nonurban neighborhoods were considered deprived (ACORN categories 4 and 5; 27.8%). Moreover, when urbanicity and neighborhood deprivation were included in a logistic regression model together, they were both significantly associated with childhood psychotic symptoms (OR = 1.62, 95% CI = 1.03–2.56, *P* = .039; OR = 1.62, 95% CI = 1.05–2.50, *P* = .029, respectively), demonstrating that urbanicity is associated with childhood psychotic symptoms largely independently of neighborhood-level deprivation in this sample. Additionally, the association between urbanicity and childhood psychotic symptoms held when earlier urbanicity at age 5 was examined (OR = 1.80, 95% CI = 1.16–2.77, *P* = .008). Therefore, the remaining analyses in this article will focus on tracing the effects of urbanicity (age-5 or age-12, where appropriate) on childhood psychotic symptoms.

### Is Urbanicity Specifically Associated With Childhood Psychotic Symptoms?

Our assumption of specificity to psychotic symptoms was tentatively supported, as associations between age-12 urbanicity and age-12 depression (OR = 1.16, 95% CI = 0.69–1.96, *P* = .571), anxiety (OR = 1.42, 95% CI = 0.95–2.12, *P* = .091) and antisocial behavior (OR = 0.93, 95% CI = 0.59–1.47, *P* = .753) were each nonsignificant, with smaller effect sizes than demonstrated for psychotic symptoms. However, given that the CIs for both depression and anxiety included the point estimate for the association between age-12 urbanicity and childhood psychotic symptoms (OR = 1.76), we cannot be sure that these associations differed significantly. Nevertheless, after simultaneous adjustment for age-12 depression, anxiety and antisocial behavior, urbanicity remained significantly associated with childhood psychotic symptoms (OR = 1.74, 95% CI = 1.15–2.65, *P* = .009), suggesting that urbanicity was independently associated with childhood psychotic symptoms in this sample. Furthermore, the associations between urbanicity and these 3 additional age-12 outcomes remained nonsignificant when they were recategorized at a lower threshold (80th percentile), suggesting that the negative findings were not due to inadequate power (results available upon request).

### Is the Association Between Urbanicity and Childhood Psychotic Symptoms Explained by Background Characteristics of Families Living in Cities?

The association between age-12 urbanicity and childhood psychotic symptoms did not appear to be explained by 3 key potential family-level confounders, namely family SES, family psychiatric history and maternal psychosis. Simultaneous adjustment for these proxy indicators of genetic and environmental risk only slightly attenuated the association between age-12 urbanicity and childhood psychotic symptoms (OR = 1.61, 95% CI = 1.04–2.51, *P* = .035).

### Are Urban Neighborhoods More Likely to Lack Social Cohesion and Social Control and Be Characterized by Disorder and Crime?

Associations of urbanicity with neighborhood-level social processes are shown in table 1. At age 5, urban neighborhoods had (ie, mothers reported) significantly lower social cohesion and social control, and significantly higher neighborhood disorder and crime victimization than nonurban neighborhoods (all *P*s < .001). Similar bivariate associations were found been urbanicity and social processes for age-12 neighborhoods (residents’ reports) (all *P*s < .001; table 1).

Associations of neighborhood-level social processes with childhood psychotic symptoms are shown in table 2. Children were significantly less likely to experience psychotic symptoms at age 12 if, at age 5, they lived in neighborhoods with higher social cohesion (*P* < .001) and higher social control (*P* = .003). In contrast, children were significantly more likely to experience psychotic symptoms at age 12 if their age-5 neighborhood was characterized by higher neighborhood disorder (*P* = .010) and higher crime victimization (*P* < .001). A comparable cross-sectional pattern was found for the associations between age-12 neighborhood-level social processes and childhood psychotic symptoms, though social control was borderline statistically significant (*P* = .050) and neighborhood-level crime victimization failed to reach conventional levels of statistical significance (*P* = .123; table 2).

### Do Neighborhood-Level Social Processes Mediate the Effect of Urban Residency on Childhood Psychotic Symptoms?

We investigated the extent that neighborhood-level social processes mediated the effect of urban residency on childhood psychotic symptoms (figure 1). Social processes were only included if they were significantly associated with both urbanicity and childhood psychotic symptoms (ie, age-12 neighborhood-level crime victimization was excluded as it was not associated with childhood psychotic symptoms at *P* < .05). Table 3 shows results as odds ratios with 95% CIs for the total (overall association), direct (the part of the overall association that is not explained by the mediator/covariates in the model) and indirect (the part of the overall association that is explained by the social process mediator in the model) effects of urbanicity on childhood psychotic symptoms. A model in which the indirect OR is equal to the total OR would indicate that the effect of the predictor on the outcome is entirely (100%) mediated by the specified mediator. Mediation model 1 is unadjusted, and Mediation model 2 is adjusted for family-level confounders (family SES, family psychiatric history, maternal psychosis) (Note: sample size and total effect ORs vary slightly within table 3 due to small numbers of children missing data on age-5 neighborhood-level social processes and/or family-level confounders. Further detail is provided in Table 3’s footnote.). Mediation model 1 shows that neighborhood-level low social cohesion at age 5 significantly mediated the effect of age-5 urbanicity on age-12 psychotic symptoms, explaining 25% of the association. Low social control and high crime victimization in the neighborhood also significantly mediated the effect of age-5 urbanicity on childhood psychotic symptoms, each explaining 13% of the association. These prospective models were somewhat supported by our cross-sectional analysis of age-12 urbanicity and age-12 social processes, in that low social cohesion once again significantly explained the largest proportion of the association between urbanicity and childhood psychotic symptoms (19%). These mediatory effects were slightly attenuated after considering family-level confounders (Mediation model 2, table 3). Nonetheless, following adjustment, neighborhood-level low social cohesion and high crime victimization at age 5 still significantly mediated the effect of age-5 urbanicity on childhood psychotic symptoms (explaining 17% and 11%, respectively). When age-5 social cohesion and crime victimization were simultaneously modeled, together they explained nearly a quarter of the effect of age-5 urbanicity on age-12 psychotic symptoms (24%: OR = 1.11, 95% CI = 1.03–1.20, *P* = .004).

## Discussion

This is the first study to investigate whether specific psychosocial features of the urban environment increase children’s risk for psychotic symptoms. Our findings add to existing knowledge in at least 3 ways. First, children living in urban neighborhoods were ~80% more likely to experience psychotic symptoms at age 12 compared to children living in nonurban neighborhoods. This association held in both prospective and cross-sectional models, and was not explained by the socioeconomic or psychiatric composition of urban families. Second, psychotic symptoms were more common among children living in neighborhoods characterized by low social cohesion, low social control, high neighborhood disorder, and where the family had been directly victimized by a crime. Our findings highlight that these neighborhood-level social processes, which are implicated in adult psychosis,^17,37,39,40,46–48^ may also be relevant to positive psychotic symptoms in childhood. Third, low social cohesion explained the largest proportion of the effect of urbanicity on childhood psychotic symptoms, regardless of reporter (17% for mother reports, 10% for resident reports), and independently of the potential family-level confounders measured in this study. Furthermore, social cohesion together with crime victimization at age-5 explained almost a quarter of the effect of age-5 urbanicity on childhood psychotic symptoms. Though we have investigated childhood psychotic symptoms as the main outcome measure, our findings regarding social cohesion and crime victimization are consistent with previous studies implicating area-level social fragmentation^39,41,47^ (or related constructs) and crime^40,46^ in adult psychosis.

A significant minority of children experience persistent psychotic symptoms and eventual clinical diagnosis.^31,32^ Furthermore, urban upbringing is highly correlated with urban adult residency.^6^ Taken together, ours and previous findings are consistent with the proposal that early-life exposure to neighborhood-level social stressors contributes to the heightened psychosis rates found in cities.^17–19,49,50^ From a child’s perspective, growing up in a crowded neighborhood characterized by insecure/nonexistent social support networks, unfriendly/unpredictable interactions between neighbors, and fear of/exposure to crime could promote psychotic symptoms in various mutually compatible ways. Prolonged exposure to neighborhood-level social stressors could dysregulate the hypothalamic–pituitary–adrenal axis,^76^ dopaminergic system,^50^ and/or neurodevelopment,^21^ increasing risk for psychotic symptoms particularly among children with genetic predisposition.^77^ A cognitive mechanism, with specific adverse neighborhood-level experiences exacerbating or providing content to emerging delusions and hallucinations^78^ could also explain why urbanicity was associated with positive psychotic symptoms but not significantly with anxiety or depression.^79^ These neighborhood effects could also be transmitted indirectly by heightening children’s exposure to family-level stress or even maltreatment. Indeed, low neighborhood-level social cohesion appears to undermine positive parenting practices.^80^ The individual-level factors and potential mechanisms leading from neighborhood-level adverse exposures to childhood psychotic symptoms now require attention. For example, children raised in urban vs nonurban neighborhoods could differ in their neurocognitive reactivity to social stress, as recently demonstrated among healthy adults.^81,82^

### Limitations

Five limitations deserve mention. First, causal inference is limited as families were not randomly selected into neighborhoods. Whilst we adjusted for a number of important proxy measures of genetic and environmental risk, various non/reverse-causal explanations remain possible. Future research with larger samples, and ideally quasi-experimental designs, are required to more persuasively rule out social selection as an explanation for these findings. The role of gene-environment correlation (eg, individuals with higher genetic risk for psychosis “drifting” into urban neighborhoods) can also now be estimated via emerging methods such as polygenic risk score analysis. Second, childhood psychotic symptoms are relatively rare, with only ~6% of children reporting symptoms in the E-Risk cohort at age 12. Our findings would benefit from replication. Third, this low base-rate also made it necessary to dichotomize urbanicity to increase power. This potentially simplified our findings, particularly given previous evidence for a dose-response urbanicity-psychosis association through the range of urbanicity. Fourth, although childhood psychotic symptoms are thought to lie on a continuum with schizophrenia,^83^ they are also associated with other psychiatric disorders in adulthood^26,32,33^ and therefore the current findings may extend beyond schizophrenia to risk for serious adult psychopathology in general. Finally, the E-Risk cohort is a twin sample, and whether findings from twin studies generalize to singletons is sometimes contested. However, the children in our study are representative of singletons for the prevalence of psychotic symptoms,^22–26^ and representative of UK families in terms of geographic and socioeconomic distribution.^57,58^

Importantly, neighborhood-level social processes did not completely explain the effect of urbanicity in our analyses. Future investigations should consider a wider range of potential social and physical neighborhood-level characteristics when testing for environmental contributions to childhood psychotic symptoms. Neighborhood-level physical exposures such as noise, light, and air pollution, as well as exposure to viral infections warrant research in relation to early psychotic symptoms. The modest mediation could also be partly attributable to measurement error entailed in the neighborhood-level social process measures. Additionally, it is possible that up to age 12 the children in our study were relatively sheltered from certain threats in their neighborhoods. Cumulative neighborhood-level exposures, from childhood, through adolescence and into adulthood, may each contribute in different ways or combine to increase risk for psychotic symptoms. It will therefore be important to investigate the contribution of neighborhood-level social processes to the emergence of psychotic symptoms in late adolescence, when many children will have experienced more direct exposure to adversity in their neighborhood.

## Conclusion

In this study, the increased risk for childhood psychotic symptoms in urban neighborhoods was explained, in part, by lower levels of social cohesion and higher levels of crime victimization operating within these neighborhoods. If these novel findings are replicated, they could support the role of exposure to neighborhood-level social stressors in the aetiology of childhood psychotic symptoms.^17,18,46^ Populations are becoming increasingly urban, and child and adolescent psychopathology represents a growing proportion of the global burden of disease.^84^ The present findings therefore underscore the emerging need to identify the social, psychological, and biological pathways leading from neighborhood-level exposures to childhood psychotic symptoms.

## Supplementary Material

Supplementary material is available at http://schizophreniabulletin.oxfordjournals.org.

## Funding

The E-Risk study is funded by the UK Medical Research Council (G1002190, G9806489). This work was supported by the Economic and Social Research Council (RES-177-25-0013, and a Multidisciplinary Studentship to J.N.); National Institute of Child Health and Development (HD061298); MQ: Transforming Mental Health (MQ14F40); Google Streetview; British Academy (SQ140024); William T. Grant Foundation; and Jacobs Foundation. C.L.O. is a Jacobs Foundation Advanced Research Fellow.

## Supplementary Material

Supplementary Data
