# Supplementary material for: Why Are Children in Urban Neighborhoods at Increased Risk for Psychotic Symptoms? Findings From a UK Longitudinal Cohort Study
Source: Schizophr Bull. 2016 May 6;42(6):1372–83. doi: 10.1093/schbul/sbw052 (PMC5049530; doi:10.1093/schbul/sbw052)
Supplement: Supplementary Data [file supp_42_6_1372__index.html]

Why are Children in Urban Neighborhoods at Increased Risk for Psychotic Symptoms? Findings From a UK Longitudinal Cohort Study — Why Are Children in Urban Neighborhoods at Increased Risk for Psychotic Symptoms? Findings From a UK Longitudinal Cohort Study — Supplementary Data 

# Why Are Children in Urban Neighborhoods at Increased Risk for Psychotic Symptoms? Findings From a UK Longitudinal Cohort Study

## Supplementary Data

Data files

- Supplementary Data - Supplementary Data
