# Supplementary material for: Why Are Children in Urban Neighborhoods at Increased Risk for Psychotic Symptoms? Findings From a UK Longitudinal Cohort Study
Source: Schizophr Bull. 2016 May 6;42(6):1372–83. doi: 10.1093/schbul/sbw052 (PMC5049530; doi:10.1093/schbul/sbw052)
Supplement: Supplementary Data [file supp_sbw052_Supplementary_Table_1.doc]

**Supplementary Table 1. *Main logistic regression analyses performed using both the ‘cluster’ command and multi-level modelling in STATA***

| **Predictor variable** | **Cluster analysis** | | | **Multi-level analysis**^a^ | | |
| --- | --- | --- | --- | --- | --- | --- |
| *Outcome variable* | OR | 95% CI | *p* | OR | 95% CI | *p* |
| **Age-12 urbanicity** |  |  |  |  |  |  |
| *Childhood psychotic symptoms* | 1.76 | [1.15, 2.68] | 0.009 | 2.12 | [1.22, 3.70] | 0.008 |
| *Childhood psychotic symptoms** | 1.61 | [1.04, 2.51] | 0.035 | 1.89 | [1.09, 3.30] | 0.024 |
| *Depression* | 1.16 | [0.69, 1.96] | 0.571 | 1.28 | [0.65, 2.51] | 0.472 |
| *Anxiety* | 1.42 | [0.95, 2.12] | 0.091 | 1.57 | [0.94, 2.62] | 0.085 |
| *Antisocial behaviour* | 0.93 | [0.59, 1.47] | 0.753 | *initial values not feasible* | | |
|  |  |  |  |  |  |  |
| **Age-5 social cohesion**  *Childhood psychotic symptoms* | 0.68 | [0.58, 0.82] | <0.001 | 0.62 | [0.49, 0.78] | <0.001 |
| **Age-5 social control**  *Childhood psychotic symptoms* | 0.75 | [0.62, 0.91] | 0.003 | 0.69 | [0.55, 0.88] | 0.003 |
| **Age-5 neighbourhood disorder**  *Childhood psychotic symptoms* | 1.26 | [1.06, 1.51] | 0.010 | 1.33 | [1.05, 1.68] | 0.017 |
| **Age-5 crime victimisation**  *Childhood psychotic symptom* | 1.40 | [1.19, 1.65] | <0.001 | 1.54 | [1.22, 1.95] | <0.001 |
|  |  |  |  |  |  |  |
| **Age-12 social cohesion**  *Childhood psychotic symptoms* | 0.77 | [0.65 - 0.90] | 0.001 | 0.69 | [0.52, 0.90] | 0.007 |
| **Age-12 social control**  *Childhood psychotic symptoms* | 0.83 | [0.69 - 1.00] | 0.056 | 0.77 | [0.59, 1.02] | 0.064 |
| **Age-12 neighbourhood disorder**  *Childhood psychotic symptoms* | 1.27 | [1.06 - 1.52] | 0.008 | 1.39 | [1.07, 1.81] | 0.013 |
| **Age-12 crime victimisation**  *Childhood psychotic symptoms* | 1.17 | [0.95 - 1.42] | 0.133 | 1.21 | [0.95, 1.55] | 0.119 |
|  |  |  |  |  |  |  |

CI, confidence interval. OR, odds ratio. *Adjusted for family socioeconomic status, family psychiatric history and maternal psychosis. ^a^ Multi-level mixed models were estimated in STATA using the XTMELOGIT command. For odds ratios, the fixed portion of the model was specified as a binomial distribution, and random intercepts were specified with an unstructured covariance matrix to account for the clustering of twins within families. Note: it was not possible to treat twins as random effects for the association between urbanicity and neighbourhood-level social processes because twin pairs correlated 100% for these measures.
